# Endophytic microorganisms from ‘Bordô’ grapes as biological control agents against *Colletotrichum* and *Botrytis*

**DOI:** 10.1007/s42770-026-01967-z

**Published:** 2026-07-08

**Authors:** Camila Iavorski Zela, Camilla Castellar, Débora de Oliveira Franco, André Luiz Graf, Renata Faier Calegario, Louise Larissa May De Mio

**Affiliations:** 1 Programa de Pós-graduação em Produção Vegetal / PGAPV, Universidade Federal do Paraná / UFPR, Curitiba, Paraná Brasil; 2 Empresa de Pesquisa Agropecuária e Extensão Rural de Santa Catarina / Epagri, Videira, Santa Catarina Brasil; 3 Departamento de Fitotecnia e Fitossanidade, Universidade Federal do Paraná, Rua dos Funcionários, Cabral, Curitiba, 1540 Paraná Brasil

**Keywords:** Microbial antagonism, Biological control, *Vitis* sp., bioinput, Microbiome

## Abstract

*Colletotrichum* spp. and *Botrytis cinerea* are the main causal agents of grape bunch rot in Brazil. Although chemical control is widely used, it has limitations, such as the selection of resistant pathogen populations and environmental concerns. Biological control emerges as a promising alternative; however, commercial products for viticulture remain limited. We hypothesized that by prospecting endophytic microorganisms from berries of *Vitis* hosts more resistant to rots, it would be possible to identify isolates with greater biocontrol potential. In this study, 52 endophytic isolates from berries of *V. labrusca* cv. ‘Bordô’ were evaluated. In vitro assays showed mycelial growth inhibition of *C. nymphaeae* and *B. cinerea* of up to 33% and 60%, respectively. Three isolates antagonistic to both pathogens were molecularly identified as AvCaPR20-VA4L (*Clavispora asparagi*), AvZmPR20-VB5B (*Zygoascus meyerae*), and AvTmPR20-PA1N (*Tatumella* sp.). In postharvest assays, the isolates achieved 31.5–73.5% control of grape ripe rot (*C. nymphaeae*) and 39.1–59.4% control of gray mold (*B. cinerea*), with AvTmPR20-PA1N showing the highest efficacy, comparable to chlorothalonil. In untreated berries, disease incidence reached 53.1% for grape ripe rot and 100% for gray mold. Next-generation sequencing of the natural berry microbiota revealed the recurrent presence of *Clavispora* and *Tatumella* in non-inoculated ‘Bordô’ vines, confirming their natural association with grape berry tissues. These findings highlight the potential of exploring microbial diversity from naturally resistant plants as a sustainable strategy for biological disease management in viticulture.

**Supplementary Information:**

The online version contains supplementary material available at 10.1007/s42770-026-01967-z.

## Introduction

Grape bunch rot limits cultivation by affecting the sensory and commercial quality of berries and derived products, such as juice and wine [1]. In Brazil, the main bunch rot diseases are grape ripe rot and gray mold, caused respectively by *Colletotrichum* spp. (Glomerellaceae) and *Botrytis cinerea* (Sclerotiniaceae) [2].

Chemical control remains the predominant strategy, but it is hampered by pre-harvest restrictions, the emergence of resistant pathogen populations, and environmental and health risks [3]. Biological control offers a sustainable alternative [4]. In Brazil, *Bacillus* and *Trichoderma* species are the most commonly used microbial agents in viticulture. Furthermore, other microorganisms, including *Paecilomyces lilacinus*, *Purpureocillium lilacinum*, and *Clonostachys rosea*, are registered for disease biocontrol in the country’s agriculture [5].

Endophytes from cultivars that remain healthy under pathogen pressure, such as ‘Bordô’ (*V. labrusca*), which may show lower susceptibility to bunch rot under certain conditions [6], are promising sources of antagonistic microorganisms. Instead of focusing only on traditional habitats such as soil, rhizosphere, or phylloplane [7], exploring grapevine endophytes may be particularly effective, especially for managing bunch rot infections that originate during flowering or remain latent in berries [8].

Endophytes inhabit internal plant tissues without causing symptoms [9] and can suppress pathogens through competition, production of antimicrobial compounds, or induction of host resistance [10], thereby contributing to reduced dependence on pesticides [11]. Endophytic isolates from leaves of ‘Concord’ and ‘Bordô’ grapevines have already shown antagonistic potential against *Alternaria*, *Colletotrichum*, and *Sphaceloma* spp [12]. The composition of these communities is influenced by factors such as geographic location, microclimate, and vineyard management practices [13], and recent molecular tools now enable comparative analyses across different environments.

The ‘Bordô’ cultivar is often considered less susceptible to bunch rots, possibly due to its earlier harvest at lower soluble solids content (13–14 °Brix) [14], compared to *Vitis vinifera* cultivars (> 20 °Brix). Since fruit maturation is associated with increased susceptibility to pathogens such as *Colletotrichum* spp [15]., this characteristic may reduce infection risk. Additionally, ‘Bordô’ berries may harbor endophytic microorganisms with biocontrol potential. Therefore, this study aimed to isolate endophytes from ‘Bordô’ berries grown under different fungicide application regimes (0, two, and five applications during the harvest), evaluate their antagonistic potential, and analyze their occurrence within the natural microbiota of the berries to guide future applications in biocontrol.

## Materials and methods

### Experimental area and sampling

Berries of *V. labrusca* cv. ‘Bordô’ were collected in the 2019/20 season from a commercial vineyard in Campo Largo, Paraná, Brazil (25°24′10.5″S; 49°29′46.7″W). Three management strategies were applied: (a) no fungicide; (b) two applications (December 19–31, 2019); and (c) five applications (November 4–December 31, 2019) of copper oxychloride, difenoconazole, and pyraclostrobin + metiram. The experimental design and the fungicides used followed the protocols described by Castellar et al. [16]. Samples at physiological ripening (i.e., ºBrix ≥ 14) were collected on Jan 20, 2020. For each treatment, 16 bunches were collected (one from each previously marked shoot), packed in thermal boxes, and transported to the laboratory for processing.

### Isolation of endophytic microorganisms

Eight berries per bunch were surface-sterilized (70% ethanol for 1 min, 0.5% sodium hypochlorite for 1 min, followed by three rinses in sterile distilled water), dried, and cut to extract 50 µL of undiluted juice per bunch, which was directly plated onto culture media. No serial dilutions were performed. Three culture media were used: Potato Dextrose Agar (PDA) supplemented with difenoconazole (12 mg L⁻¹), Nutrient Agar (NA), and Luria–Bertani (LB). Plates were incubated at 28 °C under a 12 h photoperiod. After 48 h, a single ~ 2-mm colony from each plate was transferred to liquid culture. Isolates were preserved in 25% glycerol at − 20 °C.

### Preparation of grape berry extract for endophytic microbiota analysis

Aliquots of grape juice from treatments with zero and five fungicide applications were stored at − 20 °C and subjected to metagenomic sequencing (GoGenetic^®^). For each treatment, one extract was prepared by pooling the 16 bunches collected. The two-application treatment was excluded to focus on the most contrasting conditions.

### In vitro antagonistic activity assays

The endophytes obtained from grape juice were tested in vitro against *Colletotrichum nymphaeae* (VlCnPR20-A1R2) and *Botrytis cinerea* (VvBcSP21-B1), using the dual culture method [17]. Isolates were reactivated for three days at 28 ± 2 °C on their original media (Table 1), resuspended in 1 mL of 0.85% NaCl, and 100 µL were plated and incubated for 24 h. Pathogen mycelial discs were placed at the center of PDA plates, and 5 µL of endophyte suspension was applied at four equidistant points, 15 mm from the center. Plates were incubated at 24 ± 2 °C (12 h photoperiod) and evaluated after four days for *B. cinerea* and seven days for *C. nymphaeae*. Mycelial growth inhibition (%) was calculated relative to pathogen-only controls.

**Table 1:** Endophytic isolates from *Vitis labrusca* berries obtained from areas with different disease management strategies and cultured on various media during the 2019/2020 growing season. Underlined are the isolates selected for their antagonistic potential against *Colletotrichum* and *Botrytis* in both experimental replicates

0 fungicide application			2 fungicide applications			5 fungicide applications		
PDA	LB	NA	PDA	LB	NA	PDA	LB	NA
Isolates code								
PA1B	PA1L	*PA1N*	AC3B	AA3L	AA4N	VA2B	VA1L	VA3N
PA2B	PA3L	PA2N	AC4B	AA4L	AB2N	VB3B	VA2L	VA4N
PC4B	PA4L	PB2N	-	AB2L	AC2N	*VB5B*	VA3L	VB1N
-	PB3L	PB3N	-	AC2L	AC3N	-	*VA4L*	VB2N
-	PB4L	PC2N	-	AC4L	-	-	VB2L	VB3N
-	PC2L	PC3N	-	AD3L	-	-	VC1L	VB4N
-	PD4L	PC4N	-	-	-	-	VC2L	VC1N
-	-	PD3N	-	-	-	-	VD1L	VC2N
-	-	-	-	-	-	-	VD3L	VC4N
-	-	-	-	-	-	-	-	VD3N
Number of isolates per culture medium								
3	7	8	2	6	4	3	9	10
Total number of isolates per fungicide regime								
	18			12			22	

PDA – Potato Dextrose Agar; LB – Modified Luria Bertani; NA – Nutrient Agar. Codes: before PA1N = AvTmPR20-; before VB5B = AvZmPR20-; before VA4L = AvCaPR20-

Based on growth relative to controls, interactions were classified as antagonistic (growth < control), synergistic (growth > control), or indifferent (no significant difference) (Supplementary information 1). Isolates that consistently inhibited both pathogens in both experimental repetitions were considered promising and selected for postharvest biocontrol assay and molecular identification. Each treatment had three replicates, and the experiment was conducted twice. Data were analyzed by ANOVA (F-test) (Supplementary information 2), followed by Dunnett’s test (α = 0.05), after verifying assumptions of variance homogeneity (Bartlett’s test) and residual normality (Shapiro–Wilk test), with log transformation applied when necessary.

### Postharvest biocontrol on grape berries

Grape berries of cv. ‘Thompson’ were surface-sterilized and subjected to the following treatments: the endophytic isolates AvCaPR20-VA4L, AvZmPR20-VB5B, and AvTmPR20-PA1N; the commercial biocontrol product Duravel^®^ (*Bacillus amyloliquefaciens*, 1 g L⁻¹); and the fungicide Prevenil^®^ (chlorothalonil, 50 µL L⁻¹). Sterile distilled water was used as the control. Berries were immersed for 5 s in treatment suspensions.

For treatment preparation, bacterial isolates were reactivated on NA medium and transferred (40 µL) to Erlenmeyer flasks containing liquid Nutrient Broth. Cultures were incubated at 28 °C for 24 h under agitation (120 rpm), and cell suspensions were standardized to 2 × 10⁵ CFU mL⁻¹.

Twenty-four hours after treatment, berries were inoculated with 20 µL of conidial suspension (1 × 10⁵ conidia mL⁻¹) of each pathogen. Conidia of *B. cinerea* were prepared in a 1:1 mixture of water and grape juice.

After inoculation, berries were placed on moistened filter paper inside Gerbox^®^ containers and incubated in a Conviron^®^ chamber at 23 °C with a 12 h photoperiod. A completely randomized design was used, consisting of four replicates, each containing eight berries. The entire assay was repeated once, resulting in two independent experimental runs. Daily evaluations were performed, and disease incidence was recorded when the control treatment surpassed 50% symptomatic berries, using: Incidence (%) = Symptomatic berries/Total berries×100.

Data were analyzed by ANOVA (F-test) (Supplementary information 2), followed by Tukey’s test (α = 0.05), after confirming variance homogeneity (Bartlett’s test) and residual normality (Shapiro–Wilk test).

### Molecular identification of promising endophytic isolates

After selection based on antagonistic activity, promising endophytes were identified by sequencing the ITS region (yeasts) or 16 S rRNA (bacteria). DNA was extracted using the Wizard Magnetic DNA Purification System kit (Promega^®^), amplified by PCR with primers ITS1/ITS4 [18] or 27 F/1492R [19, 20] (Supplementary information 3), and sequenced by the Sanger method on GoGenetic^®^. The consensus sequences were compared to GenBank entries via BLASTn, and deposited under accession numbers PV571904 (*Clavispora asparagi*), PV571905 (*Zygoascus meyerae*), and PV567724 (*Tatumella* sp.). Phylogenetic analyses were performed using Bayesian inference and maximum likelihood, with methodological details provided in Supplementary information 4.

### Analysis of the natural endophytic microbiota of ‘Bordô’ berries

To assess whether the promising microorganisms were abundant in berries, a metagenomic analysis of the endophytic community was performed. Total DNA was extracted with the DNeasy Power Soil kit (Qiagen^®^), and the 16 S rRNA [21] and ITS [22] regions were amplified and sequenced on the Illumina NextSeq platform. Sequence data were processed in QIIME2 v.2023.9 [23]. Taxonomic assignment was carried out using the GTDB v.207 [24] and UNITE v.9.0 databases for bacteria and fungi, respectively.

After classification, sequences assigned to Plantae (e.g., chloroplast or mitochondrial origin) were manually removed from the feature table prior to downstream analyses. Only sequences assigned to the target domains (Bacteria or Fungi) were retained, and genera with relative abundance > 0.1% were considered for community composition analyses. Alpha diversity indices (Chao1, Shannon, Simpson [25–27]) were calculated in RStudio using the phyloseqGraphTest v.0.1.1 package [28] (Supplementary information 5).

## Results

### Isolation of endophytic microorganisms from *Vitis labrusca* berries

A total of 52 endophytic isolates was obtained from *V. labrusca* cv. ‘Bordô’ berries (Table 1), and all were tested in the antagonism assays. These comprised 18 isolates from untreated berries, 12 from berries with two applications, and 22 from berries with five applications.

### In vitro interactions between *Vitis labrusca* berry endophytes and the pathogens

**Fig. 1:**
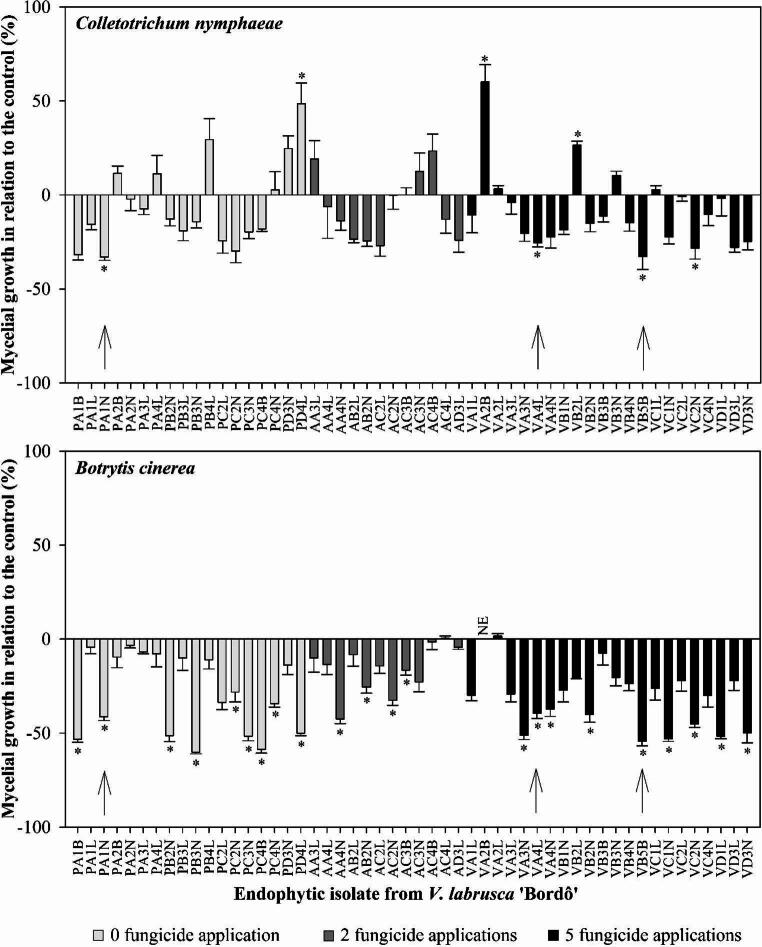
Mycelial growth (%) of *Colletotrichum nymphaeae* and *Botrytis cinerea* relative to the control for endophytic isolates from *Vitis labrusca* cv. ‘Bordô’ berries. Isolates marked with * were significantly different from the control (Dunnett’s test, α = 5%). Arrows indicate isolates selected for antagonistic potential against *Colletotrichum* and *Botrytis* in both experimental replicates. The bars represent the standard error (SE) across replicates. NE – Not evaluated. Codes: before PA1N = AvTmPR20-; before VB5B = AvZmPR20-; before VA4L = AvCaPR20-

Most isolates exhibited an antagonistic effect on pathogen mycelial growth. On average, mycelial growth in the presence of endophytes was lower than in the control (Fig. 1). The responses varied between the two pathogens tested, *C. nymphaeae* and *B. cinerea*, with no consistent effect of the disease management strategy on endophyte performance.

Among the 52 isolates tested against *C. nymphaeae*, four reduced pathogen growth (10.4–33.1%), while three promoted growth (26.6–60.2%). Against *B. cinerea*, 28 isolates reduced mycelial growth (16.6–60.1%), and none promoted it (Fig. 1).

Four isolates inhibited both pathogens in both experimental replicates: AvTmPR20-PA1N (from berries without fungicide), and AvCaPR20-VA4L, AvZmPR20-VB5B, and VC2N (from berries with five applications). Three were selected for molecular and phylogenetic analysis, based on their original isolation media: AvTmPR20-PA1N and VC2N from NA medium; the others from LB and PDA.

### Postharvest biocontrol on grape berries

The treatments showed significant differences in disease incidence in berries inoculated with *C. nymphaeae* and *B. cinerea*. Overall, the selected endophytes reduced the incidence of both diseases compared with the control (Table 2). For *C. nymphaeae*, incidence in the control was 53.1%, whereas endophytic isolates reduced this value to 14.1–37.5%. For *B. cinerea*, the control exhibited 100% incidence, and all treatments significantly reduced disease levels to 40.6–60.9%. The isolate AvTmPR20-PA1N was the most effective, with performance comparable to the fungicide chlorothalonil, followed by AvZmPR20-VB5B and AvCaPR20-VA4L. All endophytes showed results similar to the commercial biological product.

**Table 2:** Disease incidence and incubation period (IP) of grape berries inoculated with *Colletotrichum nymphaeae* and *Botrytis cinerea* after treated with grape endophytic isolates, *Bacillus amyloliquefaciens*, and chlorothalonil in postharvest assays

Treatment	Grape ripe rot		Gray mold	
	Incidence (%)	IP (day)	Incidence (%)	IP (day)
Control¹	53,1 a	10	100,0 a	2
AvTmPR20-PA1N²	14,1 cd	> 10	40,6 cd	> 2
AvCaPR20-VA4L³	37,5 ab	> 10	53,1 bc	2
AvZmPR20-VB5B^4^	25,0 bc	> 10	60,9 b	2
*B. amyloliquefaciens* ^5^	32,8 b	> 10	53,1 bc	2
Chlorothalonil^6^	3,1 d	> 10	35,9 d	> 2

¹Sterile distilled water; ²*Tatumella morbirosei*; ³*Clavispora asparagi*; ^4^*Zygoascus meyerae*; ^5^Commercial product Duravel^®^; ^6^Commercial product Previnil^®^

For *C. nymphaeae*, all treatments increased the time of appearance of the first symptoms (incubation period -IP) compared with the control. For *B. cinerea*, the IP was longer than the control (no treated fruit) only in the treatments with AvTmPR20-PA1N and chlorothalonil.

### Molecular identification of antagonistic endophytic isolates from *Vitis labrusca* berries

PCR amplification confirmed AvCaPR20-VA4L and AvZmPR20-VB5B as fungi (ITS ~ 560 bp) and AvTmPR20-PA1N as a bacterium (16 S rRNA ~ 1500 bp). BLASTn comparison of sequences identified these isolates as belonging to the genera *Clavispora*, *Zygoascus*, and *Tatumella*, respectively.

**Fig. 2:**
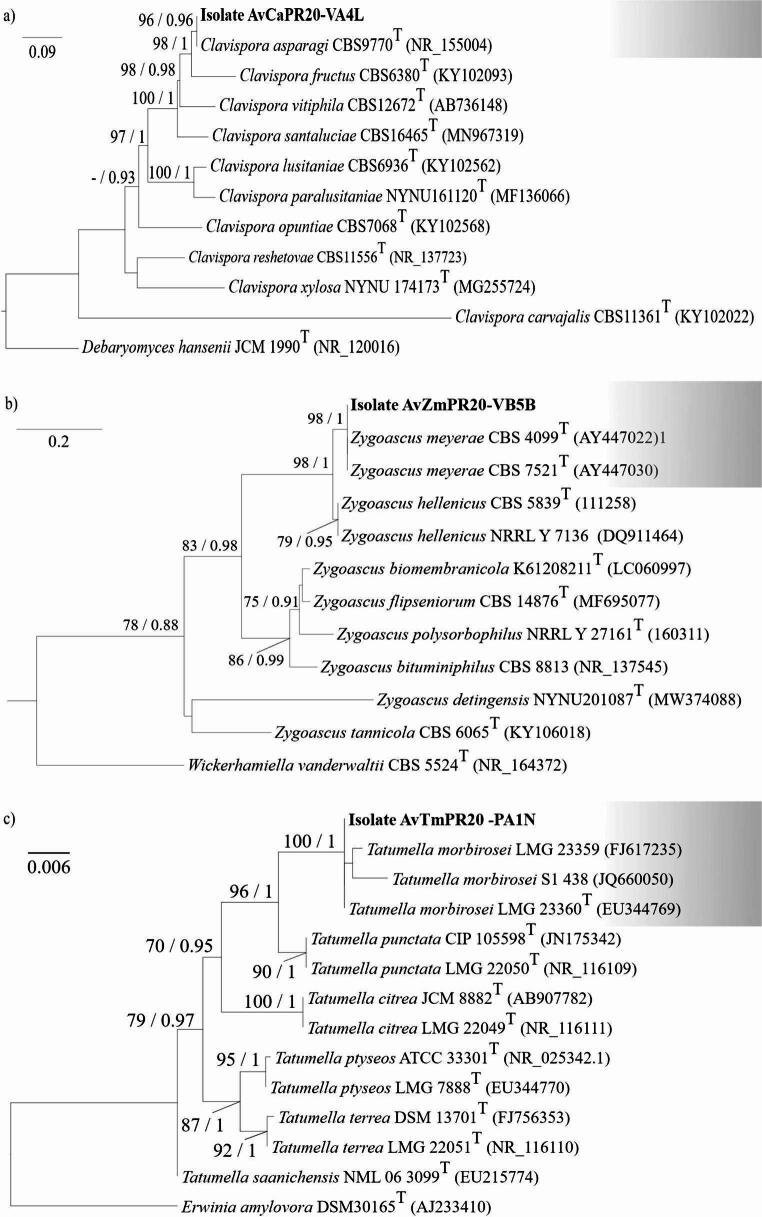
Phylogenetic trees inferred by Bayesian inference (BI) from molecular markers of selected isolates. (a) ITS region from isolate AvCaPR20-VA4L (in bold), including all accepted type species of the genus *Clavispora* (isolates marked with “T”) and the outgroup *Debaryomyces hansenii*. ML bootstrap support values above 80% and Bayesian posterior probability values (PP) above 0.9 are shown at the nodes, in that order. (b) ITS region from isolate AvZmPR20-VB5B (in bold), including all accepted type species of the genus *Zygoascus* (isolates marked with “T”) and the outgroup *Wickerhamiella vanderwaltii*. ML bootstrap support values above 75% and Bayesian posterior probability values (PP) above 0.7 are shown at the nodes, in that order. (c) 16 S rRNA gene from isolate AvTmPR20-PA1N (in bold), including all accepted type species of the genus *Tatumella* (isolates marked with “T”) and the outgroup *Erwinia amylovora*. ML bootstrap support values above 70% and Bayesian posterior probability values (PP) above 0.75 are shown at the nodes, in that order. The scale bar indicates the number of nucleotide substitutions per site

Phylogenetic analysis showed that AvCaPR20-VA4L clustered with *C. asparagi* CBS 9770 (GenBank: NR_155004), AvZmPR20-VB5B with *Z. meyerae* CBS 4099 (GenBank: KY106012), and AvTmPR20-PA1N with *T. morbirosei* LMG 23,360 (GenBank: EU344769) (Fig. 2a – c), which was supported by BLASTn identities of 99.5%, 100%, and 99.0%, respectively, to the corresponding type strains.

### Effect of fungicide application on the composition and diversity of the grape-associated microbiota

**Fig. 3:**
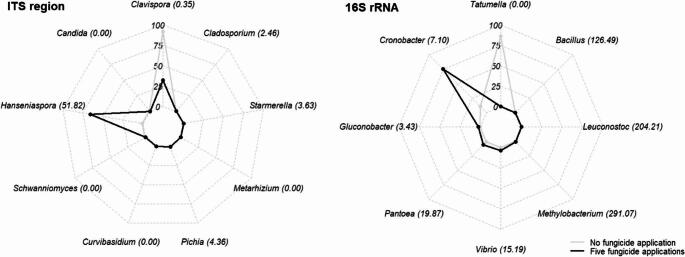
Relative abundance of endophytic fungal (ITS region) and bacterial (16 S rRNA gene) genera in grape berries under different fungicide application managements; the number in parentheses shows the proportion of the number of reads in berries without application compared to berries with 5 fungicide applications

Metagenomic sequencing of the ITS and 16 S regions revealed clear changes in the endophytic fungal and bacterial communities of *V. labrusca* berries under contrasting fungicide regimes. In untreated berries (0 applications), the fungal community was dominated by *Clavispora* (51.8%), while treated berries (5 applications) showed a marked replacement of this genus by *Hanseniaspora* (65.6%) (Fig. 3). Other fungal genera detected with an abundance above 0.1% included *Cladosporium*, *Starmerella*, *Pichia*, and *Schwanniomyces*, although all of them occurred differently between treatments: *Schwanniomyces* was detected only in untreated berries, while *Pichia* and *Starmerella* were more abundant in treated berries. No sequences corresponding to the main pathogenic genera *Botrytis* or *Colletotrichum* were detected above the established limit of 0.1%, suggesting very low levels of latent infection or efficient suppression by the endophytic community.

The bacterial community also differed markedly between treatments. *Tatumella*, one of the antagonistic isolates identified in vitro, was the most abundant genus in untreated berries (43.4%), while *Cronobacter* dominated treated berries (71.0%). Other genera included *Gluconobacter*, *Pantoea*, and *Vibrio* which also varied in abundance depending on fungicide exposure (Fig. 3). In addition, the genera *Leuconostoc*, *Methylobacterium*, and *Bacillus* were detected exclusively in berries subjected to five fungicide applications, with no reads corresponding to these taxa in untreated samples.

## Discussion

The results of this study highlight the potential of endophytic microorganisms isolated from *V. labrusca* cv. ‘Bordô’ berries for biological control of *C. nymphaeae* and *B. cinerea*, regardless isolation from grapes collected from areas with or without fungicide application. Among the 52 isolates that were able to inhibit both pathogens, 2 fungal and 1 bacterial isolate were selected. Molecular analyses identified the fungal isolates as *Clavispora asparagi* (AvCaPR20-VA4L) and *Zygoascus meyerae* (AvZmPR20-VB5B), while the bacterial isolate (AvTmPR20-PA1N) was assigned to the genus *Tatumella* based on 16 S rRNA sequence analysis.

Two postharvest assays confirmed the effectiveness of the antagonists in reducing both gray mold and ripe rot on ‘Thompson’ grapes. No commercial products based on these species are currently available, underscoring their potential as novel biocontrol agents to be used alone or in combinations. A greater number of endophytes inhibited *B. cinerea* compared to *C. nymphaeae*. In the orchard where the endophytes isolates were collected no gray mold symptoms were observed in the source orchard, while ripe rot appeared sporadically. We hypothesized that the lower antagonism against *C. nymphaeae* could be relate to its endophytic lifestyle, as *Colletotrichum* spp. are known grapevine endophytes [29], potentially conferring an adaptive advantage over those isolates.

Some endophytic isolates stimulated the growth of *C. nymphaeae*, whereas no such effect was observed for *B. cinerea*. This contrasting response indicates that interactions between endophytic microorganisms and pathogens are species-specific and may shift from antagonistic to facilitative depending on the microbial combination involved [30]. From an applied perspective, these findings have direct implications for disease management. They demonstrate that not all endophytes are suitable as biological control agents, as some may enhance the performance of specific pathogens. Therefore, screening programs for microbial biocontrol agents should not rely solely on antagonistic assays but also incorporate tests to detect potential stimulatory effects on target pathogens. This is particularly relevant in complex pathosystems, such as grapevine diseases, where multiple pathogens coexist [2]. Failure to consider such interactions may result in inconsistent disease control outcomes under field conditions.

The three selected isolates were confirmed through molecular identification. AvCaPR20-VA4L and AvZmPR20-VB5B met the ≥ 99% ITS identity threshold for fungi [31], and phylogenetic analyses confirmed their classification as *C. asparagi* and *Z. meyerae*, respectively, with strong support [31, 32]. AvTmPR20-PA1N showed 99.0% 16 S rRNA identity with *Tatumella* sp. and was placed in a well-supported clade, corroborating Brady et al. [33], who reclassified several *Pantoea* species as *Tatumella*, including *P. citrea*, explaining the presence of *Pantoea* in our tree.

*Clavispora* genus have been reported as promising biological control agents, particularly in postharvest systems, where they can suppress pathogens through competition and tolerance to environmental stresses. For example, *Clavispora lusitaniae* has demonstrated efficacy against *Penicillium digitatum*, maintaining viability and antagonistic activity under stress conditions typical of fruit storage environments [34]. In addition, species such as *C. phyllophila*, *C. vitiphila*, and *C. santaluciae* have been reported in association with grapevine tissues [35, 36], supporting their ability to persist in this environment.

*Zygoascus meyerae* has also been associated with grape ecosystems and presents attributes that support its potential as a biological control agent. In addition to its occurrence in grape-associated microbial communities [37], this species produce β-glucosidase [38] which can be related to plant defense, but this should be further investigate. Its antagonistic activity against *B. cinerea* has already been demonstrated by the production of volatile and diffusible compounds, indicating a direct role in disease suppression [39] and also by the metabolic activity influencing fermentation processes [37, 38, 40].

*Tatumella* genus are frequently detected in grape-associated microbiota [41]. Although less explored as biological control agents, their presence in asymptomatic grapes infected by *Botrytis* suggests a possible association with reduced symptom expression [42]. The high abundance of *Tatumella* observed in this study, combined with its antagonistic performance, indicates that this genus could play a relevant role in the suppression of grape pathogens being a potential biocontrol agent to be investigated.

The effectiveness of endophytic isolates obtained in this work may be linked to the cultivar ‘Bordô’, known as less susceptible to bunch rot compared to other *V. labrusca* and *V. vinifera* cultivars [6]. This trait is associated with lower sugar content (~ 15 ºBrix) [14], which may hinder pathogen development [15], but genetic background may also contribute to this behavior. Indeed, ‘Bordô’ has been used as a genetic source in the development of Brazilian cultivars such as BRS Violeta, BRS Rúbea, and BRS Carmem, contributing to traits associated with resistance to downy mildew (*Plasmopara viticola*) [43]. In addition to confirming the antagonistic activity of *C. asparagi* and *Tatumella* sp., the high relative abundance of *Clavispora* and *Tatumella* in the berry microbiota suggests that, in addition to physicochemical factors, microbial composition may contribute to host resilience. Applying these isolates to susceptible cultivars during flowering (a critical infection phase) [8] could provide a sustainable bunch rot control strategy. Future studies comparing endophytic communities among cultivars grown under the same terroir conditions and management practices may help clarify these interactions.

Preliminary studies conducted after the present screening showed that the selected isolates grow efficiently in liquid culture, reaching concentrations of up to 10¹² CFU mL⁻¹ within the first 24 h of incubation, and formulation studies are currently underway. In addition, assays on grape leaves and berries did not reveal pathogenic effects when the isolates were applied individually or as a consortium (data not shown). Nevertheless, additional studies are required before practical application, including detailed biosafety assessments, pathogenicity tests in animals and humans, evaluation of formulation strategies, validation of disease control efficacy under greenhouse and vineyard conditions. Overall, the microbiota associated with *V. labrusca* ‘Bordô’ presents promising resources for bunch rot management and highlights the relevance of exploring microbial biodiversity for sustainable viticulture.

## Supplementary Information

Below is the link to the electronic supplementary material.

Supplementary Material 1 (DOCX 771 KB)

## Data Availability

The datasets generated and analyzed during this study are available from the corresponding author upon request.
