# Supplementary material for: Endophytic microorganisms from ‘Bordô’ grapes as biological control agents against Colletotrichum and Botrytis
Source: Braz J Microbiol. 2026 Jul 8;57(1):198. doi: 10.1007/s42770-026-01967-z (PMC13346379; doi:10.1007/s42770-026-01967-z)
Supplement: Supplementary file 1 — Supplementary Material 1 (DOCX 771 KB) [file 42770_2026_1967_MOESM1_ESM.docx]

**Endophytic microorganisms from *Vitis labrusca* cv. ‘Bordô’ berries inhibit *Botrytis* and *Colletotrichum* and are naturally abundant in grape tissues**

Camila Iavorski Zela¹, Camilla Castellar², Débora de Oliveira Franco¹, André Luiz Graf Júnior¹, Renata Faier Calegario¹, Louise Larissa May De Mio¹

**Supplementary information 1**

Fig. S1. Morphological aspects of the colony of isolate VlCnPR20-A1R2 (*Colletotrichum nymphaeae*) after pairing with endophytic isolates from *Vitis labrusca* cv. ‘Bordô’ berries. (A) Control or endophytic isolates showing no effect on pathogen growth; (B) Antagonistic interaction between berry endophytic isolates and the mycelial growth of *C. nymphaeae*; (C) Synergistic interaction between berry endophytic isolates and the mycelial growth of *C. nymphaeae*.


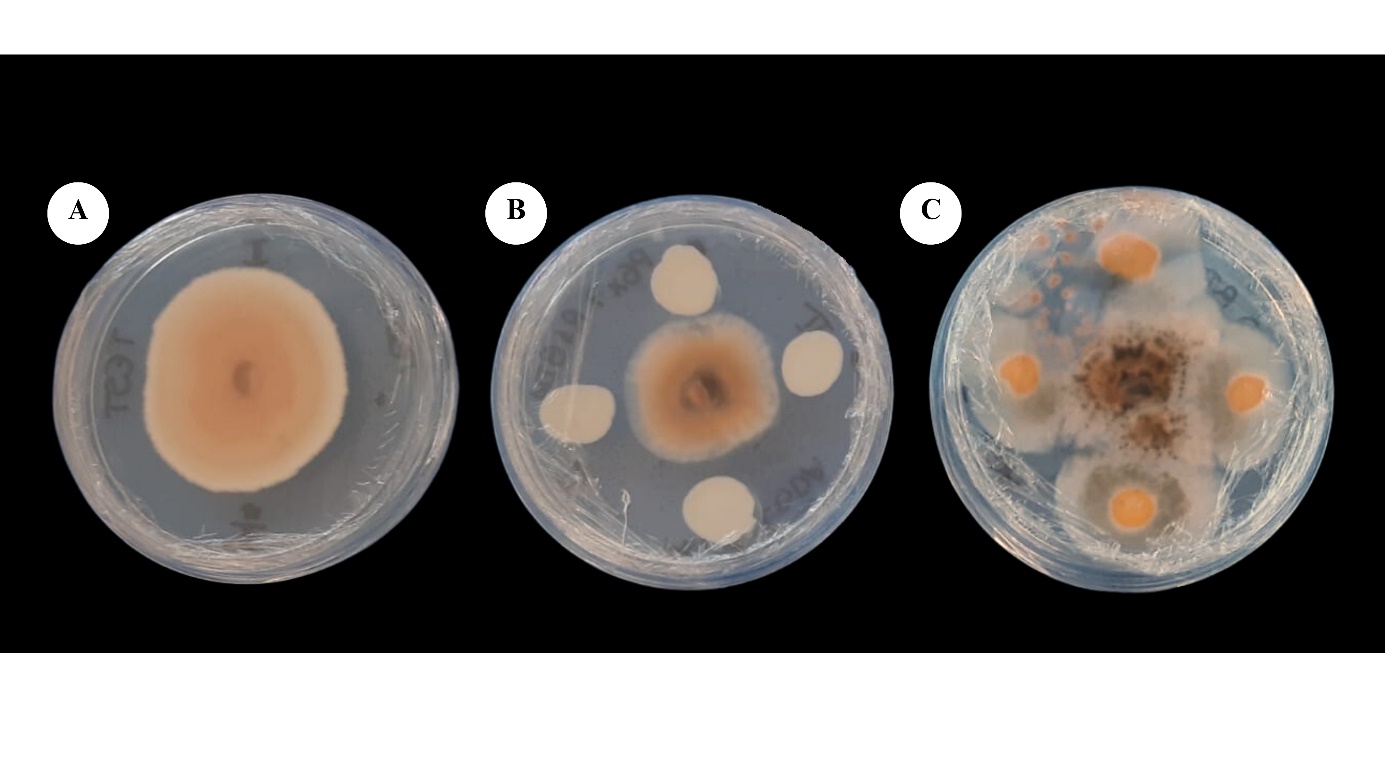


**Supplementary information 2**

Table S1. Analysis of variance (ANOVA) results for inhibition of *Colletotrichum nymphaeae* and *Botrytis cinerea* by different endophytic isolates from *Vitis labrusca* ‘Bordô’ *in vitro*. Data are presented as F-values with degrees of freedom and P-values, with analyses performed separately for each assay.

| **Pathogen** | **Assay** | **df (treatment; residual)** | **F-value** | **P-value** |
| --- | --- | --- | --- | --- |
| *Colletotrichum nymphaeae* | 1a | (8 ; 18) | 20.7 | <0.001 |
|  | 1b | (12 ; 26) | 5.7 | <0.001 |
|  | 1c | (10 ; 22) | 7.8 | <0.001 |
|  | 1d | (22 ; 46) | 24.8 | <0.001 |
|  | 2a | (8 ; 18) | 33.5 | <0.001 |
|  | 2b | (12 ; 26) | 14.8 | <0.001 |
|  | 2c | (10 ; 22) | 4.6 | <0.001 |
|  | 2d | (22 ; 46) | 11.4 | <0.001 |
| *Botytis cinerea* | 1a | (7 ; 16) | 66.3 | <0.001 |
|  | 1b | (22 ; 46) | 7.1 | <0.001 |
|  | 1c | (22 ; 46) | 11.2 | <0.001 |
|  | 2a | (7; 16) | 30.9 | <0.001 |
|  | 2b | (22 ; 46) | 14.0 | <0.001 |
|  | 2c | (22 ; 46) | 13.6 | <0.001 |

Table S2. Analysis of variance (ANOVA) results for the incidence of symptoms caused by *Colletotrichum nymphaeae* and *Botrytis cinerea* on grape berries during postharvest assays. Data are presented as F-values with degrees of freedom and P-values, with analyses performed separately for each pathogen.

|  | *Botrytis cinerea* | *Colletotrichum nymphaeae* |
| --- | --- | --- |
| **df(treatment; assay; residual)** | (5 ; 1 ; 41) | (5 ; 1 ; 41) |
| **F-value (treatment; assay)** | (46.55 ; 1.31) | (17.56 ; 0.37) |
| **P-value (treatment; assay)** | (<0.0001 ; 0.26) | <0.0001; 0.54 |

**Supplementary information 3**

Table S3. Primers used for amplification of genomic DNA from *Vitis labrusca* endophytic and pathogenic isolates. ^¹^ITS = Internal Transcribed Spacer; GAPDH = Glyceraldehyde 3-phosphate dehydrogenase; TUB2 = β-tubulin 2; G3PDH = Glyceraldehyde 3-phosphate dehydrogenase; 16S = Ribosomal RNA. Sequences marked with * represent species-specific primers.

| **Isolate** | **Gene or region** | **Primer** | **Reference** |
| --- | --- | --- | --- |
| AvCaPR20-VA4L | ITS | ITS1 (5'-TCCGTAGGTGAACCTGCG G-3') | White *et al.* [1] |
|  |  | ITS4 (5'-TCCTCCGCTTATT GATATGC-3') |  |
| AvZmPR20-VB5B | ITS | ITS1 (5'-TCCGTAGGTGAACCTGCG G-3') | White *et al.* [1] |
|  |  | ITS4 (5'-TCCTCCGCTTATT GATATGC-3') |  |
| AvTmPR20-PA1N | *16S* | 27F (5’-AGTTTGATCCTGGCTCAG-3’) | Lane [2] |
|  |  | 1492R (5’-GGTTACCTTGTTACGACTT-3’) | Stackebrandt; Liesack [3] |

**Supplementary information 4**

Genomic DNA from the isolates was extracted using the Wizard Magnetic DNA Purification System kit (Promega®), following the manufacturer's instructions. The PCR reactions were performed with a final volume of 25.0 uL, adding 12.5 uL of GoTaq® G2 Colorless Master Mix (Promega®), 1.0 uL of each primer forward and reverse (10 mM), 7.5 uL of free nuclease H2O and 3.0 uL of DNA (10 ng/L). The reactions were carried out in the Thermo Scientific Arktik Thermal Cycler (Thermo Fisher Scientific Inc.) with the following settings: 4 min at 96 ºC for initial denaturation, 35 cycles at 96 ºC for 30 s, 60 ºC for 45 s and 72 ºC for 90 s, ending with 72 ºC for 5 min. The amplified fragments were visualized by electrophoresis on 1% (wt/vol) agarose gels, purified and sequenced using the Sanger method by GoGenetic®. The consensus sequences were processed in SeqAssem (v.07/2008) [4] and compared to sequences available in the GenBank/NCBI database (National Center for Biotechnology Information) using BLASTn (https://blast.ncbi.nlm.nih.gov/Blast.cgi). Multiple alignments were performed with MAFFT v.7 online [5] and manually adjusted with MEGA 7 [6]. Phylogenetic trees were constructed using Bayesian inference (IB) approach, with the MrBayes v.3.2 plugin [7] and maximum likelihood (ML), with the IQ-TREE plugin [8]. Evolutionary models were defined by ModelFinder [9], based on the corrected Akaike criterium. For BI, the trees were sampled every 10,000 generations, and posterior probability value were calculated after discarding the initial 25% of sampled trees as burn-in. The analysis was performed using two parallel runs, each comprising one cold and three heated chains, and continued until the average standard deviation of split frequencies reached ≤ 0.01. For ML analyses, branch support values were estimated using the ultrafast bootstrap method and the SH-aLRT branch test based on 1000 replicates. The resulting trees were visualized using FigTree v.1.4.4 [10] and edited in Inkscape (www.inkscape.org/). The selection of reference sequences was based on data from LPSN (List of Prokaryotic names with Standing in Nomenclature) (https://lpsn.dsmz.de/) [11] for bacteria, and MycoBank (https://www.mycobank.org/) for fungi and yeasts (Table S3). The species selected as outgroups for the phylogenetic trees were: *Debaryomyces hansenii* CBS 767 (GenBank: NR_120016) for the genus *Clavispora* [12]; *Erwinia amylovora* DSM 30165 (GenBank: AJ233410) for *Tatumella* [13]; and *Wickerhamiella vanderwaltii* CBS 5524 (GenBank: NR_164372) for *Zygoascus* [14].

Table S4. Isolates used for phylogenetic analysis of endophytic microorganisms from *Vitis labrusca* berries with antagonistic potential.

| **Species** | **Code** | **Source** | **Location** | **ITS*** |
| --- | --- | --- | --- | --- |
| *Clavispora asparagi* | NRRL Y-48714^T^ = CBS 9770^T^ | *Asparagus filicinus* fruit | China | NR_155004 |
| *Clavispora carvajalis* | NRRL Y-48694^T^ = CBS 11361^T^ | Rotten wood | Ecuador | KY102022 |
| *Clavispora fructus* | NRRL Y-17072^T^ = CBS 6380^T^ | Unknown | Japan | KY102093 |
| *Clavispora lusitaniae* | CBS 6936^T^ | *Citrus* essence | Israel | KY102562 |
| *Clavispora opuntiae* | NRRL Y-11820^T^ = CBS 7068^T^ | Cactus | Australia | KY102568 |
| *Clavispora paralusitaniae* | NYNU161120^T^ | Rotten wood | China | MF136066 |
| *Clavispora phyllophila* | CBS 12671^T^ | *V. vinifera* leaf | Thailand | Not available |
| *Clavispora reshetovae* | CBS 11556^T^ | Pasture soil | Germany | NR_137723 |
| *Clavispora santaluciae* | A1.5^T^ = A_1.18^T^ = CBS 16465^T^ | *V. labrusca* grape | Portugal | MN967319 |
| *Clavispora vitiphila* | CBS 12672^T^ | *V. vinifera* leaf | Thailand | AB736148 |
| *Clavispora xylosa* | NYNU 174173^T^ | Rotten wood | China | MG255724 |
| *Debaryomyces hansenii* | CBS 767^T^ = JCM 1990^T**^ | Crab | Brazil | NR_120016 |
| *Wickerhamiella vanderwaltii* | CBS 5524^T**^ | Unknown | Unknown | NR_164372 |
| *Zygoascus biomembranicola* | K61208211^T^ | Tomb of Kitora | Japan | LC060997 |
| *Zygoascus bituminiphilus* | CBS 8813^T^ | Tar | Canada | NR_137545 |
| *Zygoascus detingensis* | NYNU201087^T^ | Rotten wood | China | MW374088 |
| *Zygoascus flipseniorum* | CBS 14876^T^ | Soil | Netherlands | MF695077 |
| *Zygoascus hellenicus* | CBS 5839^T^ | Bovine udder mastitis | Germany | 111258 |
| *Zygoascus meyerae* | CBS 4099^T^ | Fermenting grape must | Greece | AY447022 |
| *Zygoascus ofunaensis* | NRRL Y-10998^T^ | Soil | Japan | Not available |
| *Zygoascus polysorbophilus* | NRRL Y 27161^T^ | White oil and polysorbate emulsion | South Africa | 160311 |
| *Zygoascus tannicola* | CBS 6065^T^ | Tanning fluid | Spain | KY106018 |
| *Erwinia amylovora* | DSM 30165^T**^ | Pear | United Kingdom | AJ233410 |
| *Tatumella citrea* | JCM 8882^T^ | Tangerine | Japan | AB907782 |
| *Tatumella citrea* | LMG 22049^T^ | Tangerine | Japan | NR_116111 |
| *Tatumella morbirosei* | LMG 23360^T^ | Pineapple | Philippines | EU344769 |
| *Tatumella morbirosei* | LMG 23359 | Pineapple | Philippines | FJ617235 |
| *Tatumella morbirosei* | S1 438 | Jatropha | China | JQ660050 |
| *Tatumella ptyseos* | LMG 7888^T^ | Human | United States | EU344770 |
| *Tatumella ptyseos* | ATCC 33301^T^ | Human | United States | NR_025342 |
| *Tatumella punctata* | CIP 105598^T^ | Tangerine | Japan | JN175342 |
| *Tatumella punctata* | LMG 22050^T^ | Tangerine | Japan | NR_11610 |
| *Tatumella saanichensis* | NML 06-3099^T^ | Human | Canada | EU215774 |
| *Tatumella terrea* | DSM 13701^T^ | Soil | Japan | FJ756353 |
| *Tatumella terrea* | LMG 22051^T^ | Soil | Japan | NR_116110 |

* GenBank accession number (<https://www.ncbi.nlm.nih.gov/>) ** Isolate used as outgroup

**Supplementary information 5**

Alpha diversity analyses (Chao1, Shannon, Simpson) revealed contrasting patterns among communities (Fig. S2). For fungi, Chao1 richness decreased after fungicide exposure, while Shannon and Simpson indices increased, indicating that although the number of taxa decreased, community evenness increased under intensive chemical management. For bacteria, all indices were higher in treated berries, suggesting an enrichment in richness and diversity under repeated fungicide applications. These results collectively indicate that fungicide use alters fungal and bacterial communities in distinct ways, reducing fungal richness and promoting bacterial proliferation and diversification.

Fig. S2. Alpha diversity indices of the endophytic microbiota associated with grape berries, assessed using the ITS region (fungi) and 16S rRNA gene (bacteria), comparing samples under different fungicide application managements. Values are descriptive; no inferential tests were applied due to lack of biological replication.


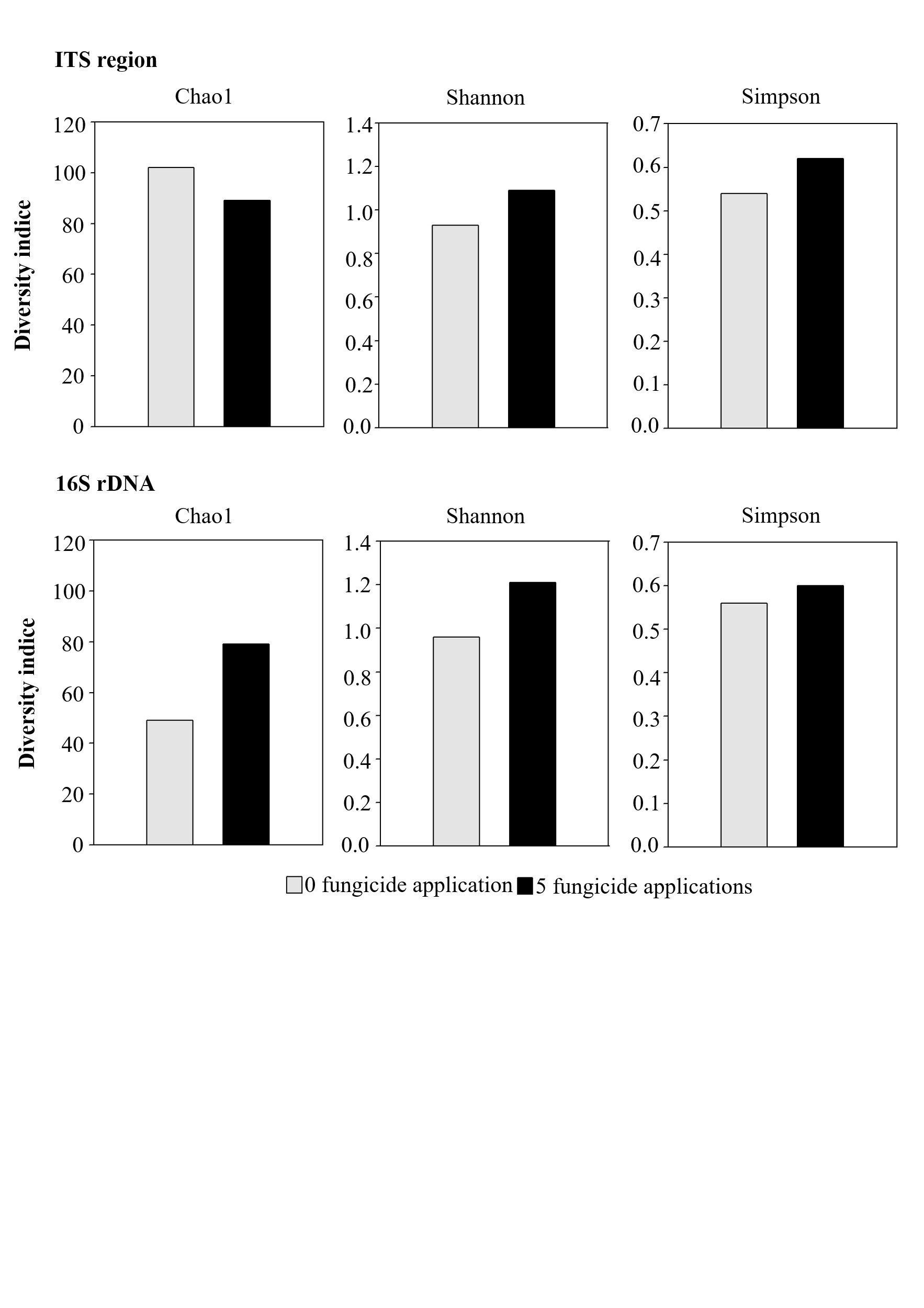


**References**

1. White TJ et al. (1990) Amplification and direct sequencing of fungal ribosomal RNA genes for phylogenetics. In: Innis MA, Gelfand DH, Sninsky JJ, White TJ (eds) PCR protocols: a guide to methods and applications. Academic Press, San Diego, pp 315–322. <https://doi.org/10.1016/B978-0-12-372180-8.50042-1>

2. Lane DJ (1991) 16S/23S rRNA sequencing. In: Stackebrandt E, Goodfellow M (eds) Nucleic acid techniques in bacterial systematics. John Wiley & Sons, New York, pp 115–175

3. Stackebrandt E, Liesack W (1993) Nucleic acids and classification. In: Goodfellow M, O'Donnell AG (eds) Handbook of new bacterial systematics. Academic Press, London, pp 152–189

4. Hepperle D (2004) SeqAssem: a sequence analysis tool contig assembler and trace data visualization tool for molecular sequences. Win32-Version. <http://science.do-mix.de>

5. Katoh K, Rozewicki J, Yamada KD (2019) MAFFT online service: multiple sequence alignment, interactive sequence choice and visualization. Brief Bioinform 20(4):1160–1166. <https://doi.org/10.1093/bib/bbx108>

6. Kumar S, Stecher G, Tamura K (2016) MEGA7: Molecular Evolutionary Genetics Analysis version 7.0 for bigger datasets. Mol Biol Evol 33:1870–1874. <https://doi.org/10.1093/molbev/msw054>

7. Ronquist F et al (2012) MrBayes 3.2: efficient Bayesian phylogenetic inference and model choice across a large model space. Syst Biol 61:539–542. <https://doi.org/10.1093/sysbio/sys029>

8. Nguyen LT et al. (2015) IQ-TREE: a fast and effective stochastic algorithm for estimating maximum-likelihood phylogenies. Mol Biol Evol 32(1):268–274. <https://doi.org/10.1093/molbev/msu300>

9. Kalyaanamoorthy S et al. (2017) ModelFinder: fast model selection for accurate phylogenetic estimates. Nat Methods 14(6):587–589. <https://doi.org/10.1038/nmeth.4285>

10. Rambaut, A (2018) FigTree v.1.4.4. <http://tree.bio.ed.ac.uk/software/figtree>

11. LPSN: List of Prokaryotic Names with Standing in Nomenclature. <http://www.bacterio.net/>

12. Inderbitzin P, Robbertse B, Schoch CL (2020) Species identification in plant-associated prokaryotes and fungi using DNA. Phytobiomes J 4:101–112. <https://doi.org/10.1094/PBIOMES-12-19-0067-RVW>

13. Brady CL et al. (2010) Transfer of *Pantoea citrea*, *Pantoea punctata* and *Pantoea terrea* to the genus *Tatumella* emend. as *Tatumella citrea* comb. nov., *Tatumella punctata* comb. nov. and *Tatumella terrea* comb. nov. and description of *Tatumella morbirosei* sp. nov. Int J Syst Evol Microbiol 60(3): 751–758. <https://doi.org/10.1099/ijs.0.012070-0>

14. Groenewald M et al. (2018) Diversity of yeast species from Dutch garden soil and the description of six novel Ascomycetes. FEMS Yeast Res 18(7). <https://doi.org/10.1093/femsyr/foy076>
